# Acknowledgement to Reviewers of *Veterinary Sciences* in 2017

**DOI:** 10.3390/vetsci5010004

**Published:** 2018-01-12

**Authors:** Veterinary Sciences Editorial Office

**Affiliations:** MDPI AG, St. Alban-Anlage 66, 4052 Basel, Switzerland

Peer review is an essential part in the publication process, ensuring that *Veterinary Sciences* maintains high quality standards for its published papers. In 2017, a total of 65 papers were published in the journal. Thanks to the cooperation of our reviewers, the median time to first decision was 29 days and the median time to publication was 67 days. The editors would like to express their sincere gratitude to the following reviewers for their time and dedication in 2017:
Akbar, SaminaLegge, MelissaAnderson, Kevin L.Leroy, BaptisteAndré-Fontaine, GenevieveLiesegang, AnnetteArmand, MartineLinder, DeborahArtemiou, ElpidaLitman, MarcusAvery, Anne C.Lloyd, JaniceAvery, PaulLuciano, FernandoBacon, HeatherLymberopoulos, Dimitrios K.Bai, HuaLyons, Leslie A.Bangoura, BeritMackenstedt, UteBarua, AnimeshMadeira De Carvalho, Luis ManuelBaviera-Puig, AmparoMadison-Antenucci, SusanBaviskar, PradyumnaMalissiova, EleniBennett, PeterMarrs, CarlBertelloni, FabrizioMartini, MinaBidarimath, MallikarjunMatthew, SusanBielefeldt-Ohmann, HelleMayser, Peter AndreasBinfet, John TylerMcBride, AnneBosilevac, MickMeadows, ShannonBoswood, AdrianMena, IgnacioBoudreau, BethMenzies, PaulaBowles, DougMexas, Angela MarieBuch, ShilpaMilting, HendrikCadenas, EnriqueMohamed Hafez, HafezCaires, KyleMoore, Robert J.Call, Douglas R.Morgan, StewartCallanan, SeanMueller, KristinaCambier, CaroleMueller, Megan KielyCannon, ClaireMurphy, BrianCannon, Claire M.Murugaiyan, JayaseelanCarlisle, Gretchen K.Narute, PurushottamCarlsson, CatharinaNeerukonda, SabarinathCarretón, ElenaNelson, Richard WCatry, BoudewijnNelson, Richard W.Chahory, SabineNermes, MerjaChandler, MarjorieNilsson, AkeChen, GuiqianNoli, ChiaraChiara, GiudiceNorton, NadineCho, Jeong-ilO’Handley, RyanChowdhury, ShafiqulÖdman, AnnaCianciolo, RachelOhara, TakahiroCOLAVITA, GiampaoloOmboni, StefanoCooke, BarbaraOn, StephenCork, SusanOverall, Karen L.Dell, ColleenOwen, HelenDiaz, DuartePackman, WendyDiesel, Alison BPaltrinieri, SaverioDima, CristianPassalaqua, KarlaDurham, Amy C.Passantino, GiuseppeDuscher, Georg G.Pathania, RajneeshEconomou, VangelisPerrucci, StefaniaEisler, MarkPinna, CarloElder, John H.Pirrone, FedericaEliades, TheodorePlesch, Petra NicoleEmmott, EdwardPoglayen, GiovanniEvans, MeirionPolakof, SergioFadok, ValeriePoli, AlessandroFawcett, AnnePonzio, PatriziaFei, Andrew Chang-YoungRamos, VictoriaFerri, MaurizioRäsänen, Pekka TuomasFlecknell, PaulReddy, SakamuriFletcher, JonReinhard-Bjornvad, CharlotteFölster-Holst, ReginaRessel, LorenzoFoote, Sally J.Roberta, PeregoFouladkhah, AliyarRoosje, PetraFravalo, PhilippeSakai, HirokiFrench, AnneSakwe, AmosGaluppi, RobertaSantovito, ElisaGay, NoellieSanz, IvanGhosh, AnuradhaSaxena, VikasGilding, Edward K.Shen, ZhenyuGilger, Brian C.Shepherd, M. L.Giulivi, CeciliaSheppard, Mary N.Gordon, Catherine A.Shil, NirajGraham, PeterShu, SherryHales, Dale BuchananSingh, Ravinder J.Han, ZheStefanon, BrunoHassan, Sharifah SyedSteinbach, Sarah M. L.Hayette, Marie-PierreStiles, JeanHeinze, Cailin R.Stone, DianaHelmy, Yosra A.Suravajhala, PrashanthHerrel, AnthonyTaboada, EduardoHezzell, MelanieTeske, ErikHildebrandt, NicolaiThomas, DaveHildreth, BlakeTidona, FlavioHodgson, KateTidwell, AmyHoenig, MargaretheTonolo, GiancarloHowell, TiffaniTvarijonaviciute, AstaHumer, ElkeUpadhyaya, InduJaja, IshmaelVan Wettere, ArnaudJang, Young DalVerbalis, JosephJohnson, Gayle C.Verstegen, Martin W. A.Jones, Meinir G.Vitale, MariaJones, PhilipVitztum, ColeyKang, Ji-HounVorbruggen, GerdKeane, Orla M.Waal, Theo DeKent, Michael S.Westman, MarkKetzis, Jennifer K.White, Amelia G.Kim, Kyung-suWhite, Stephen DavidKong, WeiliWiederstein-Grasser, IrisKostomitsopoulos, Nikolaos G.Wynwood, Sarah J.Kuhne, FranziskaYang, Vicky K.Kumar, AsitYang, ZhengyuLabella, AlejandroZandvliet, M.M.J.M. (Maurice)Larsen, JenniferZhu, GuoqiangLarsen, Jennifer A.Zienius, DainiusLashley, Victoria

